# Hv1 channel in immune cells and pharmacology

**DOI:** 10.1016/j.phrs.2025.107885

**Published:** 2025-07-26

**Authors:** Liang Yan, Jianhua J. Liu, Liang Hong

**Affiliations:** a Department of Medicine, University of Illinois Chicago, Chicago, IL 60612, USA; b Department of Pathology, University of Illinois Chicago, Chicago, IL 60612, USA; c Department of Physiology and Biophysics, University of Illinois Chicago, Chicago, IL 60612, USA; d Department of Biomedical Engineering, University of Illinois Chicago, Chicago, IL 60612, USA

**Keywords:** Hv1, Immune cells, Inflammation, Immune disorder, Ion channel, Pharmacology

## Abstract

The voltage-gated proton channel Hv1 is a key regulator of immune cell function, crucial for maintaining pH homeostasis, supporting reactive oxygen species (ROS) production, and modulating both innate and adaptive immune responses. By facilitating proton extrusion in response to membrane depolarization, Hv1 helps counteract intracellular acidification during immune cell activation and respiratory burst. Hv1 is highly expressed in diverse immune populations such as neutrophils, monocytes, eosinophils, and B cells, where it plays important roles in microbial defense, inflammatory signaling, antigen presentation, and cellular activation. Emerging evidence shows that dysregulation of Hv1 expression or function alters immune homeostasis and contributes to the pathogenesis of autoimmune diseases, chronic inflammation, and cancers. Given its immunoregulatory functions and disease associations, Hv1 represents a promising therapeutic target for the development of novel treatments for immune and inflammatory diseases.

## 1. Introduction

**Fig. 1.**
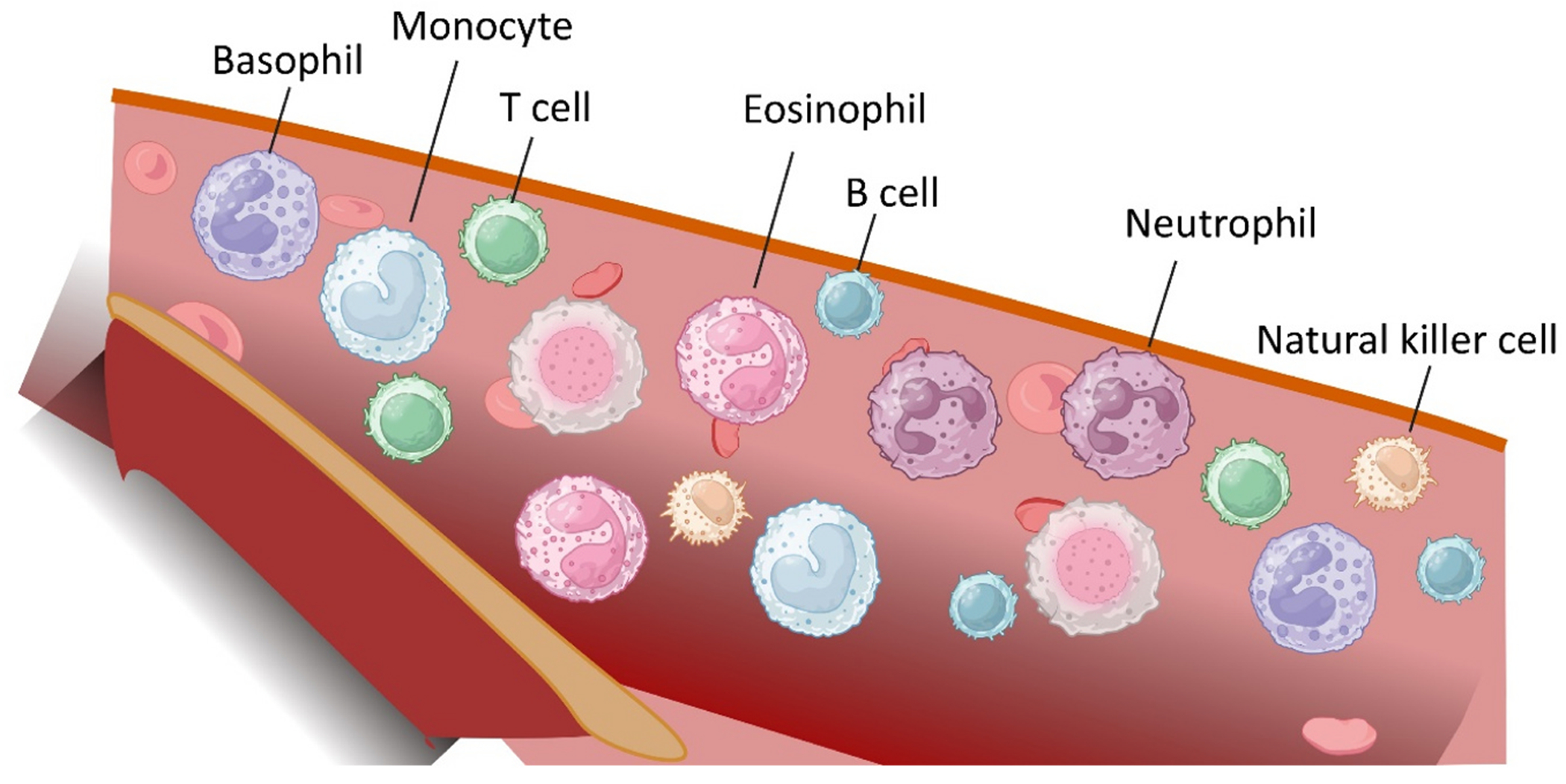
Immune cells in the blood. Immune cells are categorized into two major groups: innate and adaptive immune cells. Innate immune cells provide rapid, nonspecific responses, including neutrophils, basophils, eosinophils, monocytes, and natural killer cells. Adaptive immune cells generate highly specific and long-lasting immunity, including B and T cells.

Immune cells are the key components of the immune system, responsible for defending the body against infections, eliminating damaged or malignant cells, and maintaining tissue homeostasis [1–3]. These cells are divided into two main groups: innate and adaptive immune cells. Innate immune cells, such as neutrophils, macrophages, dendritic cells, and natural killer (NK) cells, provide rapid, nonspecific responses. And adaptive immune cells, including T and B lymphocytes, generate highly specific and long-lasting immunity (Fig. 1). The regulation, activation, and communication of immune cells are critical for the immune system, and dysregulation of immune cells leads to a variety of diseases, including immunodeficiency, autoimmunity, and cancer.

**Fig. 2.**
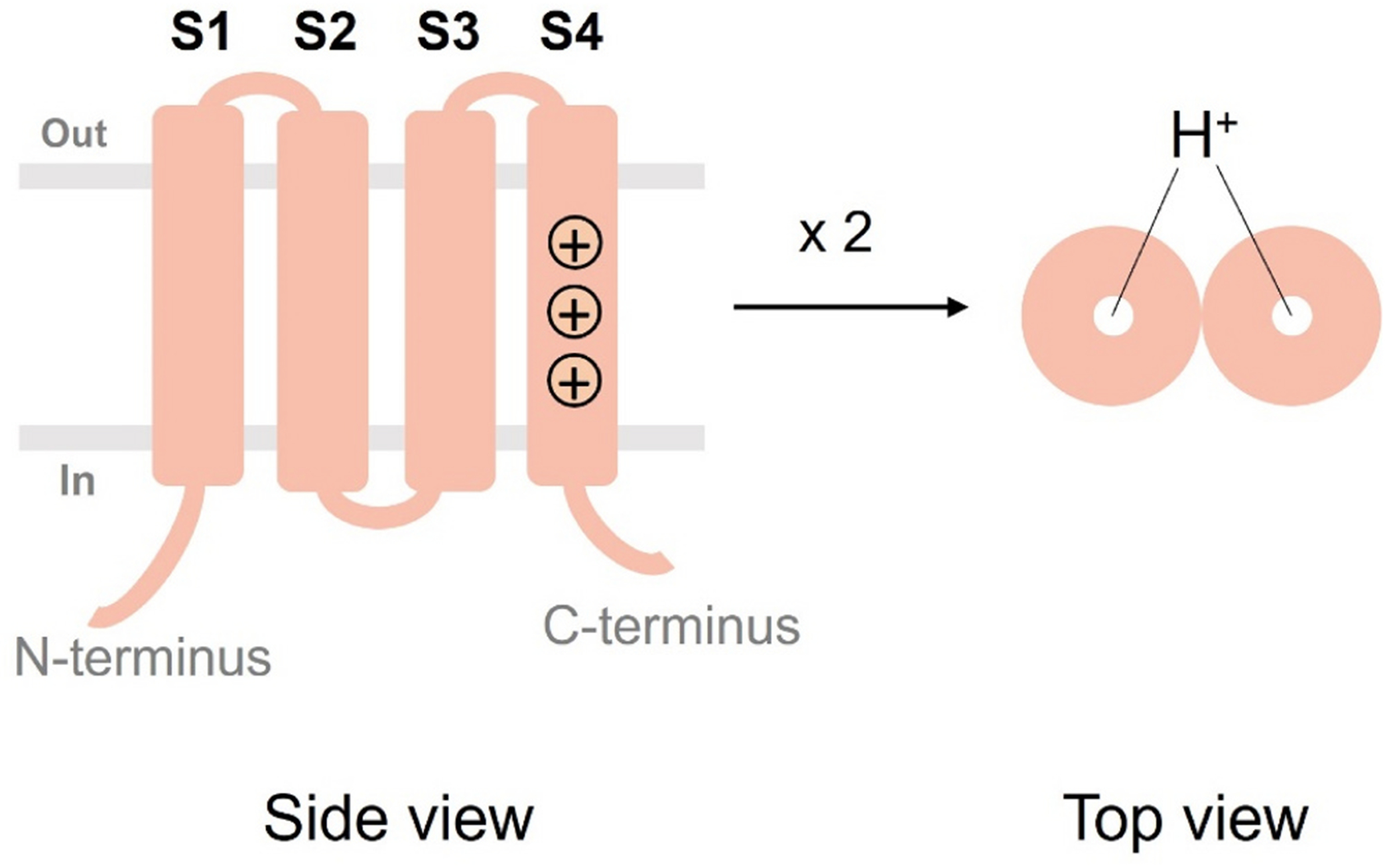
Topological organization of the voltage-gated proton channel Hv1. The Hv1 is made of four membrane-spanning segments (S1 through S4). In the S4 segment, there are three positive arginines (R205, R208, and R211 in human Hv1) responsible for the S4 movement to regulate the opening of the Hv1 proton channel. Hv1 forms dimers in which two Hv1 subunits are held together by the C-terminal coiled-coil domain of each subunit. The proton permeation pathway is located in each subunit.

The voltage-gated proton channel Hv1 (also called HVCN1) is highly expressed in various immune cells [4–8]. Hv1 is a member of the family of voltage-gated ion channels. The Hv1 channel contains a voltage-sensing domain (VSD) consisting of four membrane-spanning segments, named S1 through S4 [9–13]. The Hv1 S4 segment contains three positively charged residues (namely R205, R208, and R211 in human Hv1) responsible for the gating charge movement across the membrane electric field and opening the proton pore of the channel. Hv1 forms dimers in which two Hv1 subunits are held together by the C-terminal coiled-coil domain of each subunit. The proton permeation pathway is located in each subunit (Fig. 2).

The general role of the Hv1 proton channel is to mediate proton (H^+^) extrusion from cells and regulate cellular pH [14–17]. In immune cells, Hv1 plays a critical role in maintaining pH homeostasis and regulating reactive oxygen species (ROS) production, particularly through its interaction with the NADPH oxidase (NOX) complex.

**Fig. 3.**
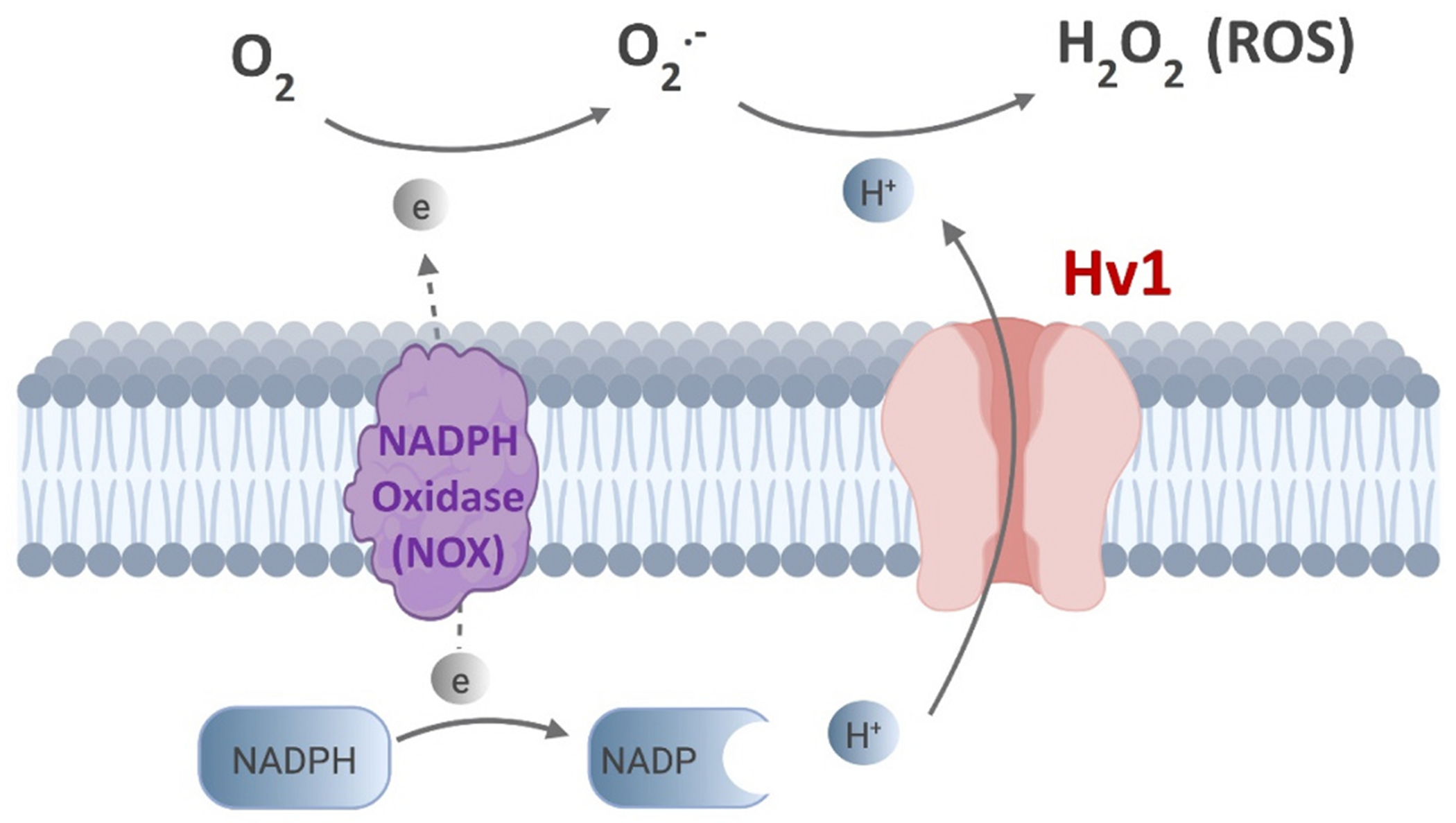
Hv1 channel controls the activity of NADPH oxidase (NOX) and drives the production of ROS, see text for more details.

ROS are a group of molecules produced in the cell through the metabolism of molecular oxygen (O_2_), such as O_2_^−^ and H_2_O_2_. The NOX enzyme uses NADPH as an electron donor [NADPH -> NADP^+^ + H^+^ + 2e^−^] and O_2_ as an electron acceptor to produce superoxide anion radical O_2_^−^ [O_2_ + e^−^ -> O_2_^−^]. The activity of the NOX results in an accumulation of protons (H^+^) in the cytosol and depolarizes the membrane which hinders the function of NOX. Hv1 proton channel extrudes these protons (i.e., Hv1-mediated H^+^ current) to repolarize the membrane which drives the activity of NOX to produce more O_2_^−^. Meanwhile, the protons extruded by Hv1 are used in the reactions of the generation of ROS from O_2_^−^ [O_2_^−^+ 2 H^+^ -> H_2_O_2_] (Fig. 3). By facilitating charge compensation during the oxidative burst, Hv1 supports the efficient generation of ROS necessary for inflammation and immune responses [5]. ROS is the driver of both physiological and pathological processes [18]. At the physiological level, ROS is a by-product of O_2_ reduction, essential for cellular signaling. ROS plays roles in the immune defense against external pathogens and acts as a second messenger to mediate the synthesis of cellular structures like protein complexes. At the pathological level, however, ROS is harmful. The elevated levels of ROS (known as oxidative stress) play crucial roles in many conditions by promoting inflammation, apoptosis, and abnormal immune responses.

Recent studies showed that Hv1 in immune cells has been linked to inflammation and immune responses. Understanding the regulatory mechanisms and functional consequences of Hv1 activity in immune cells not only provides insight into fundamental immunological processes, but also offers potential therapeutic targets for inflammatory and immune diseases.

## 2. Hv1 and immune cells

**Fig. 4.**
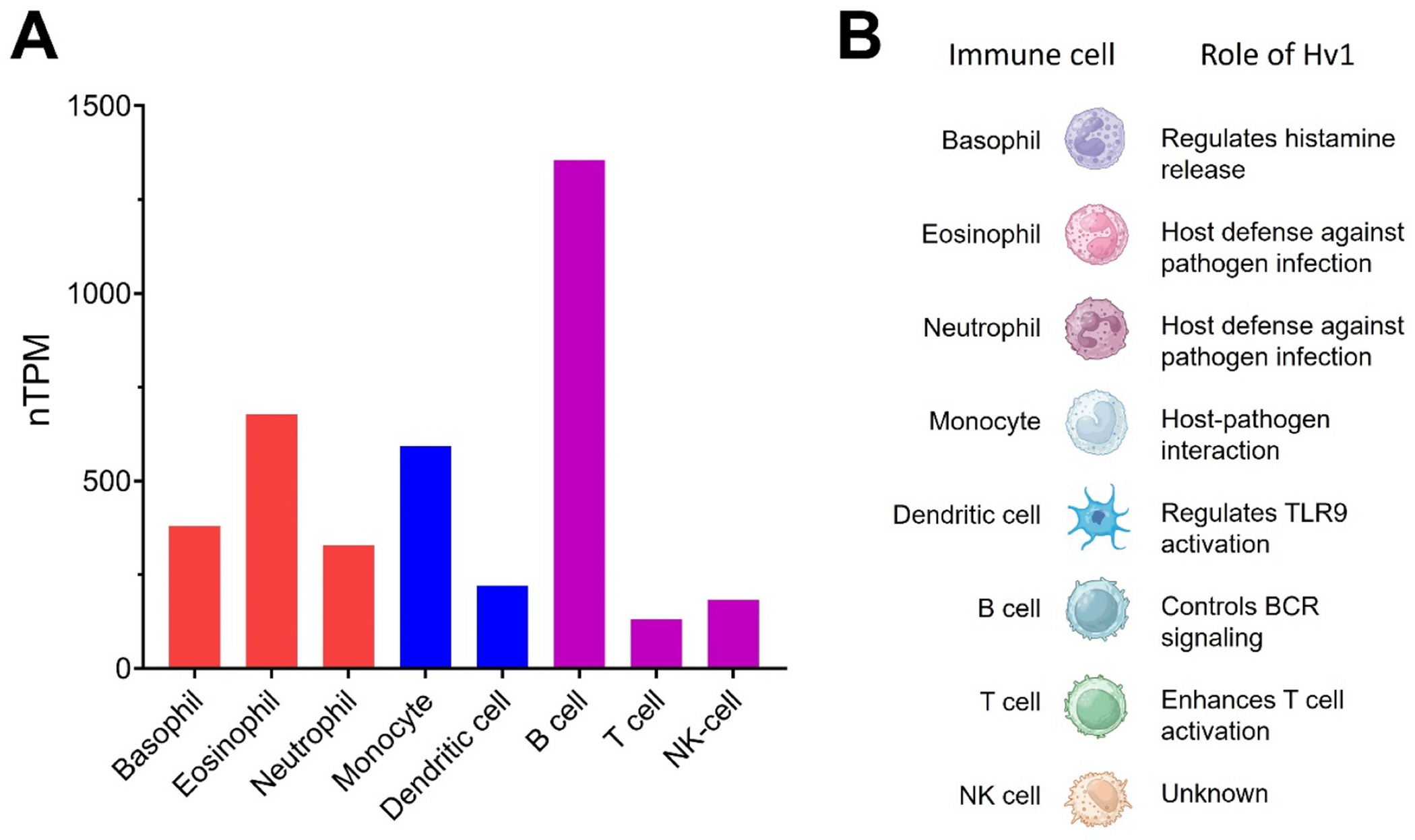
Hv1 and immune cells. (A) Expression of Hv1 in immune cells. Blood cell type expression overview shows RNA-seq data from the Human Protein Atlas (HPA) data. The resulting transcript expression values calculated as nTPM (normalized Transcripts Per Million). (B) Function of Hv1 in immune cells.

Both innate and adaptive immune cells express Hv1 channels. The human protein atlas (HPA) dataset (Human Protein Atlas proteinatlas.org) showed that Hv1 is predominantly expressed in B lymphocytes, eosinophils, monocytes, neutrophils, basophils, and dendritic cells, in which Hv1 regulates B-cell receptor (BCR) signaling, host-pathogen interaction, histamine release, and Toll-like receptor (TLR) activation (Fig. 4).

### 2.1. Hv1 in lymphocytes

#### 2.1.1. B cell

B lymphocytes play an essential role in the adaptive immune system and the production of antibodies. The maturation and activation of B cells are driven by signaling through the B-cell receptor (BCR). Hv1 channel activity controls BCR signaling and regulates B cell function [19,20]. In resting B cells, protein tyrosine phosphatases such as SHP-1 function as critical negative regulators of BCR signaling. Upon antigen engagement, the BCR is co-localized with the voltage-gated proton channel Hv1 and NADPH oxidase complex (NOX) [19,20]. The NOX transfers electrons from NADPH across the membrane to molecular oxygen, generating superoxide anions (O_2_^−^). The protons (H^+^) extruded by Hv1 channel are used in the reactions of the generation of H_2_O_2_ and other ROS from O_2_^−^. A well-characterized target of H_2_O_2_ is SHP-1, which becomes functionally inactivated through oxidative modification. In the absence of SHP-1 activity, the BCR is activated. Hv1 plays a key role in this process by supplying the H^+^ facilitating ROS generation to amplify BCR signaling, which initiates downstream BCR signaling cascades, ultimately promoting full B cell activation [19,20]. Additionally, Hv1 was implicated in B-cell malignancies [21–23]. Proteomic analysis of B-cell plasma membranes from patients with mantle cell lymphoma (MCL) identified Hv1 as a key modulator of BCR signaling underlying the development of MCL. Inhibition of Hv1 function could potentially be a therapeutic approach for B-cell malignancies that rely on sustained BCR signaling [21]. Moreover, a shorter isoform of Hv1, which lacks the first 20 amino acid residues, is enriched in malignant B lymphocytes from patients with chronic lymphocytic leukemia (CLL). B cells expressing this truncated isoform exhibit enhanced proton currents along with increased proliferative and migratory capacity [22]. These properties suggest that Hv1 may play a role in the pathogenesis of BCR-dependent B-cell malignancies. Hv1 is associated with lymphoma, and its mutations affect the B-cell receptor signaling pathway in follicular lymphoma [23].

#### 2.1.2. T cell

T lymphocytes are important components of the immune system, playing a critical role in defense against infections, allergens, and malignancies. T cells recognize antigens presented by major histocompatibility complex class I (MHCI) or class II (MHCII) molecules - CD8-positive (CD8^+^) cytotoxic T lymphocytes recognize MHCI, while CD4-positive (CD4^+^) helper T lymphocytes recognize MHCII. It was reported that *Hv1*-deficient mice displayed an increased number of activated T cells, including both CD4^+^ and CD8^+^ subsets [24]. This phenotype became more pronounced following infection with lymphocytic choriomeningitis virus. Further analysis revealed that *Hv1*-deficient T cells have significantly reduced late-phase O_2_^−^ production, indicating impaired ROS generation. These findings suggest that the absence of Hv1 impairs ROS production in T cells, which may contribute to heightened T cell activation and the development of autoimmune features [24]. Reactive nitrogen species, particularly peroxynitrite (ONOO^−^), can be generated in the tumor microenvironment via the reaction of superoxide (O_2_^−^) with nitric oxide (NO). ONOO^−^ has been shown to induce oxidative modifications, such as tyrosine nitration, on components of the T-cell receptor (TCR) complex. These modifications can alter TCR conformation and impair its signaling capacity, contributing to T-cell anergy or dysfunction, particularly in pathological contexts such as cancer [25]. This mechanism represents a key pathway through which tumors may evade immune surveillance by rendering T cells non-responsive to antigenic stimulation. Recent study showed that loss of *Hv1* resulted in heterogeneous metabolic adaptation to intracellular acidification by T cells [26], and *Hv1*-deficient CD4^+^ and CD8^+^ T cells exhibited distinct differences in their transition from naive to primed states. The differences include alterations in T cell receptor (TCR) signaling, cell division, and survival. *Hv1*-deficient naive CD4^+^ T cells undergo metabolic reprogramming to preserve glycolytic activity; however, naive CD8^+^ T cells, which express high levels of Hv1 in mitochondria, compensate for mitochondrial dysfunction in part through AMP-activated protein kinase (AMPK) activation. These findings indicate a differential role of Hv1 activity in the regulation of CD4^+^ and CD8^+^ T cell function. Hv1 activity is also associated with T-cell leukemia [27–30]. The inhibition of Hv1 by Zn^2+^ and ClGBI induced the acidification of leukemic Jurkat T cells and promoted cell apoptosis [28]. On the other side, overexpression of Hv1 in chimeric antigen receptor expressing (CAR) T cells was shown to reduce intracellular acidification and improve the antitumor activity of the CAR T cells [30].

### 2.2. Hv1 in granulocytes

#### 2.2.1. Neutrophil

Among immune cells, Hv1 has been most extensively studied in neutrophils [31–42]. Neutrophils are the most abundant of circulating immune cells and serve as first responders to infection and inflammation. Neutrophils play a central role in host defense through their capacity to phagocytose and eliminate invading pathogens, particularly bacteria. The most clearly established function of voltage-gated Hv1 proton channels in neutrophils is to sustain NOX-dependent production of ROS, which are essential for the destruction of phagocytosed bacteria [33]. Hv1 channels maintain pH in human neutrophils during phagocytosis [32], and regulate azurophilic granule release by modulating the accumulation of positive charge at the plasma membrane associated with NADPH oxidase activation [36]. Moreover, Hv1 channels extrude H^+^ and provide charge compensation for NOX activity, sustaining Ca^2+^ influx signals that regulate neutrophil adhesion and motility, *Hv1*-deficient neutrophils exhibit impaired migration *in vitro*, linked to disrupted Ca^2+^ influx after activation. This defect occurs because the absence of Hv1 causes H^+^ accumulation in the cytoplasm, which alters the driving force for Ca^2+^ entry. Impaired Ca^2+^ entry can affect downstream signaling and cytokine production, ultimately impacting neutrophil migration [38]. In addition to plasma membrane, Hv1 expresses phagosomal membranes in neutrophils [43,44], and regulates phagosomal acidification in neutrophils. The absence of Hv1 results in a more alkaline phagosomal pH, suggesting a role of Hv1 in phagosomal pH homeostasis. The Hv1 function was proposed to eliminate bacteria by maintaining pH homeostasis and ROS production in phagosomes of neutrophils [31]. Hv1 is known to promote ROS generation; however, recent evidence indicates that it can also act as a negative regulator of ROS production in neutrophils [36]. Hv1 suppresses hypochlorous acid (HOCl) production by modulating degranulation, thereby mitigating inflammation during fungal infections [36]. Additionally, it inhibits ROS-dependent activation of extracellular signal-regulated kinase (ERK) signaling [35]. These findings indicate that Hv1 might be a dual-function regulator of ROS in neutrophils.

#### 2.2.2. Eosinophil

Eosinophils are granulocytic leukocytes in allergic responses and host defense against helminthic infections. Functional Hv1-mediated proton currents in eosinophils have been characterized [45–48], and the electrophysiological properties of the Hv1 proton channels and their regulation support that they participate in H^+^ extrusion during the respiratory burst [49,50]. The Hv1-mediated proton currents control the NOX-related electron currents to modify the production of ROS [51,52], which regulates the function of eosinophils in host defense against pathogen infections. Study reported that Hv1 expression is upregulated in mouse lung eosinophils following intranasal administration of *Aspergillus fumigatus* extract [53]. Like neutrophils, eosinophils express the NOX complex. Although the expression of NOX components was similar, the amount of Hv1 was 10-fold greater in eosinophils compared to neutrophils [39]. Hv1 proton channels in eosinophils are critical for maintaining the activity of NOX, thereby supporting sustained ROS production. *Hv1*-deficient eosinophils exhibited approximately a 50 % reduction in the production of ROS [53]. However, unlike neutrophils, *Hv1*-deficient eosinophils displayed normal migratory capacity both *in vitro* and *in vivo*, as well as normal calcium signaling. Interestingly, *Hv1*-deficient eosinophils were more prone to PMA-induced cell death, likely due to their impaired ability to counteract membrane depolarization and cytosolic acidification resulting from increased NOX2 activity.

#### 2.2.3. Basophil

Basophils are non-phagocytic granulocytes involved in immediate hypersensitivity reactions. Basophils are crucial for IgE-dependent allergic responses. In most granulocytes, Hv1 proton channel activity is functionally coupled to the NOX complex. However, basophils do not express NOX, suggesting that the function of Hv1 in these cells is independent of NOX function. Despite the absence of NOX, basophils display high levels of Hv1 proton channel activity [54]. A previous study demonstrated that proton channels are activated in basophils during IgE-mediated responses, where they serve to prevent excessive intracellular acidification, potentially modulating histamine release [54]. It was shown that basophils undergo significant acidification upon activation, a process normally counteracted by proton channel activity. Notably, other pH-regulating mechanisms, such as the Na^+^/H^+^ exchanger, are insufficient to maintain pH homeostasis when Hv1 proton channels are inhibited. However, the exact influence of altered intracellular pH on degranulation and histamine release remains to be fully understood. Moreover, whether Hv1 proton channel inhibition would impact allergic responses remains to be elucidated.

#### 2.2.4. Mast cell

Mast cells are resident cells of tissue that contain many granules rich in histamine and heparin. They are issue-resident immune cells and play central roles in defending against infections and allergic reactions. The voltage-gated proton currents have been characterized in mouse bone marrow-derived mast cells and were shown to contribute to intracellular pH homeostasis of the cells [55]. Many functions of mast cells are dependent on intracellular pH (pH_i_). For instance, exocytosis of the mast cells is influenced by pH_i_ levels, and the activity of enzymes such as chymases is highly dependent on intracellular pH. Therefore, Hv1 proton channel-mediated H^+^ currents likely regulate the function of mast cell in allergic responses.

### 2.3. Hv1 in monocytes and macrophages

#### 2.3.1. Monocyte

Monocytes are essential immune cells that play a key role in protecting the body from infection and injury, as well as maintaining immune homeostasis. They contribute to both innate and adaptive immune responses. Monocytes are the largest type of leukocyte in the blood and can differentiate into macrophages in tissues, which are vital for activating adaptive immunity. Functional Hv1-mediated proton currents were characterized in human monocytes [56,57]. Monocyte Hv1 was recently identified as a cellular target for the LukAB variants [58]. LukAB is a highly polymorphic pore-forming toxin, produced by *Staphylococcus aureus*, that is highly polymorphic and can be cytotoxic to human phagocytes. Studies found that LukAB toxins target human Hv1, and *Staphylococcus aureus* kills leukocytes in a LukAB- and Hv1-dependent manner. Hv1 mediates LukAB binding and cytotoxicity, and LukAB targeting of Hv1 promotes *Staphylococcus aureus* pathogenesis [58]. This study reveals a new function of Hv1 in immune cells and highlights the role of monocyte Hv1 in host-pathogen interactions.

#### 2.3.2. Macrophage

Macrophages differentiate from monocytes when entering tissues. Macrophages function as innate immune cells through phagocytosis and sterilization of bacteria and play a central role in protecting the host from infection. In addition to clearing pathogens, macrophages present antigens to T cells to initiate the adaptive immune response and secrete cytokines that recruit immune cells and modulate inflammatory processes. Functional expressions of the Hv1 channel in macrophages have been characterized for decades [37,59,60], however, the role of macrophage Hv1 in human diseases was recently investigated. Hv1 was reported to mediate phagocytosis of apoptosis by macrophages in autoimmune disease [61]. In patients with systemic lupus erythematosus (SLE) carrying the H90 variant, the H90 variant induced lower Hv1-dependent acidification and decreased production of ROS associated with defective efferocytosis of macrophages, which led to impaired maturation and proteolysis of phagosome, and promoted kidney damage [61]. Another study reported that macrophage Hv1 is linked with the development of hypertension, and Hv1 in peritoneal macrophage acts as a sodium sensor and promotes superoxide production in a rat model of salt-sensitive hypertension [62,63].

#### 2.3.3. Microglia

Microglia are the resident macrophage-like immune cells of the central nervous system (CNS), serving as the primary defense against pathogens and injury in the CNS. They are essential for brain development and the maintenance of neural homeostasis. Microglia express the voltage-gated proton channel Hv1, which plays critical roles in ROS production, acid regulation, and neuron-microglia communication [64–67]. Hv1 has been implicated in neurological diseases such as ischemic stroke and spinal cord injury [64,68]. *Hv1*-deficiency mice showed significant protection against NOX-mediated neuronal death and brain injury 24 h following ischemic stroke [64]. The findings suggest that Hv1-mediated ROS generation contributes substantially to early brain damage after stroke and highlight Hv1 as a potential therapeutic target for ischemic stroke intervention [64,66,69]. Moreover, the expression of Hv1 is regulated in animal models of spinal cord injury (SCI), and *Hv1* knockout mice exhibited significantly improved locomotor function and reduced histopathology [68, 70–72]. Additionally, microglial Hv1 has been linked with the regulation of microenvironment in demyelination and implicated in the immunomodulation in myelin repair in the nervous system [73]. Hv1 regulates microglial M1/M2 polarization in neurological disorders [74]. M1 microglia are known to be pro-inflammatory, contributing to neurotoxicity and tissue damage, while M2 microglia are anti-inflammatory and neuroprotective, promoting tissue repair. Deficiency of *Hv1* was shown to shift the microglial polarization from M1 toward M2 state after stroke, highlighting a role of Hv1 in the modification of inflammation in neurological diseases [74].

#### 2.3.4. Osteoclast

Osteoclasts are innate immune cells within the bone, originating from the monocyte-macrophage lineage. As specialized bone-resorbing cells, they play a central role in bone remodeling [75]. Osteoclasts can be formed from tissue-specific macrophages in inflammatory or immunological conditions. Hv1-mediated proton currents were identified in mouse osteoclasts, and Hv1 activity was reported to regulate osteoclastogenesis [76–79]. Hv1 channels serve as an essential mechanism for maintaining pH homeostasis in osteoclasts, which are exposed to drastic change in pH environments during the bone resorption cycle [76]. Notably, the intracellular pH (pH_i_) affects the activity of Hv1 channel. The increase of pH_i_ in osteoclasts decreases the number of Hv1 channels available at the plasma membrane by promoting dynamin-dependent endocytosis [77]. Osteoclasts express NOX and are capable of the production of oxidative burst, the relationship between Hv1 function and NOX activity in osteoclasts is not well understood. Studies showed that extracellular phosphates enhance activities of Hv1 channels and production of ROS in murine osteoclasts [80]. However, the precise physiological role of Hv1-mediated ROS in osteoclast function remains to be elucidated.

#### 2.3.5. Kuffer cell

Kupffer cells, the liver-resident macrophages, are the largest population of tissue-resident macrophages in the body. The function of Hv1 in Kupffer cells is associated with hepatic glucose metabolism [81]. *Hv1*-deficient mice altered the hepatic oxidative stress, and exhibited elevated blood glucose and serum insulin levels despite maintaining normal insulin sensitivity, indicating that these metabolic changes are not attributable to insulin resistance. Transcriptomic analysis of *Hv1*-deficient livers revealed upregulation of genes in gluconeogenesis, indicating enhanced hepatic glucose production [81]. These findings suggest that Hv1 in Kupffer cells is a key mediator of hepatic oxidative stress and hepatic glucose metabolism. On the other hand, Kupffer cells are known to be essential in initiating and mediating immune responses to bacterial infection of the liver. However, the role of the Hv1 channel in regulating host-pathogen interaction in Kupffer cells remains unknown; further studies are required to determine the mechanism of Hv1-mediated immune responses in Kupffer cells.

### 2.4. Hv1 in other immune cells

#### 2.4.1. Dendritic cell

Dendritic cells (DCs) are key antigen-presenting cells responsible for initiating T lymphocyte activation. Upon stimulation with Toll-like receptor (TLR) ligands, such as lipopolysaccharide (LPS), DCs undergo maturation accompanied by enhanced production of reactive oxygen species (ROS) via the NOX2 complex. Hv1 plays a critical role in regulating antigen processing and presentation in dendritic cells [82,83]. An electrophysiological study demonstrated that the cytoplasmic acidification induced by NOX2 activity in mouse DCs is counteracted by proton extrusion through the Hv1 proton channel [82]. Subsequent study reveals that Hv1 controls TLR9 activation in plasmacytoid dendritic cells [83]. Hv1 activity is required for effective type I IFN responses following recognition of foreign DNA by endosomal TLR9. The deletion of *Hv1* impaired plasmacytoid DC activation in response to CpG oligonucleotides both *in vitro* and *in vivo*, resulting in reduced IFN-α secretion and diminished expression of IFN-stimulated genes. *Hv1* deficiency led to delayed endosomal acidification and increased intracellular ROS, which together compromised protease activity and TLR9 signaling. The findings establish Hv1 as a critical regulator of innate immune function in DCs and highlight its potential as a therapeutic target for modulating type I IFN–mediated autoimmune diseases.

#### 2.4.2. Myeloid-derived suppressor cell

Myeloid-derived suppressor cells (MDSCs) are a heterogeneous population of cells with potent immunosuppressive activity. They are immature myeloid cells that accumulate within the tumor microenvironment, where they play a key role in promoting immunosuppression and facilitating tumor progression. In the tumor microenvironment, MDSC-mediated immunosuppression is largely driven by their production of ROS. The MDSC-mediated ROS interacts with the T cell receptor complex, impairing antigen recognition and thereby preventing effective immune-mediated elimination of tumor cells [84]. The voltage-gated proton channel Hv1 is functionally expressed in MDSCs [84–86], and its activity is responsible for the ROS-mediated immunosuppression mechanism in MDSCs. Studies found that the N-terminal region is essential for the Hv1 channel activity in MDSCs [86]. Given its key role in supporting ROS-dependent immunosuppressive activity, Hv1 represents a promising therapeutic target. Inhibiting Hv1 may reduce MDSC-mediated immunosuppression within the tumor microenvironment, potentially enhancing anti-tumor immune responses and facilitating tumor cell clearance by the immune system [84].

## 3. Hv1 channel inhibitors with therapeutic potential

**Fig. 5.**
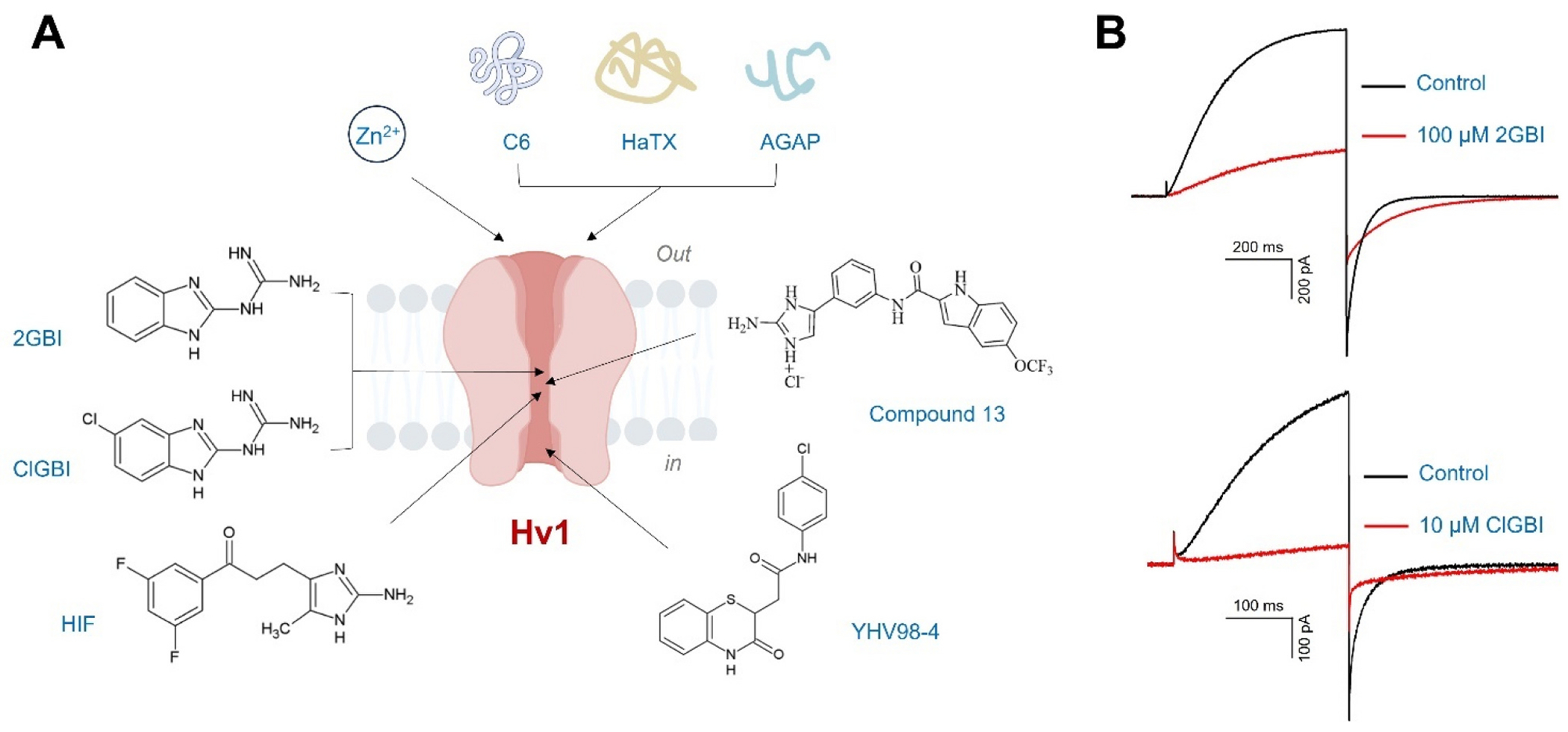
Hv1 inhibitors. (A) Zn^2+^, small-molecule inhibitors, and venom-derived peptides are identified to inhibit Hv1 function. The binding sites of guanidine derivatives are located at the core of the Hv1 channel, and the docking predicts that Compound 13 also binds the core of the channel. Zn^2+^ and peptide inhibitors target the extracellular sides of the Hv1, and YHV98–4 interacts with Hv1 at the intracellular side of the channel. The binding sites of PNX52429 and PNX61442 are not identified. (B) Functional inhibition of the Hv1 channel by guanidine derivatives. H^+^ currents measured in an inside-out patch from a *Xenopus* oocyte expressing human Hv1 channel in response to a depolarization to + 120 mV from a holding potential of −80 mV. The black trace was recorded in the absence of inhibitor, and the red trace was recorded after the addition of either 100 μM 2GBI (top panel) or 10 μM ClGBI (bottom panel) in the bath solution. pH_i_ = pH_o_ = 6.0.

Hv1 channel plays an essential role in the regulation of pH homeostasis and ROS production immune cells. Dysregulation of Hv1 has been involved in the pathophysiological implications. Increased activity of Hv1 was reported to enhance inflammation and immune responses, and H^+^ efflux mediated by Hv1 channel in immune cells was proposed to increase the production of ROS, the elevated levels of ROS (i.e., oxidative stress) are associated with many immune disorders. As discoveries linking Hv1 proton channel to immune diseases continue to grow, Hv1 represents an attractive therapeutic target in immune disorder and inflammation. Several Hv1 inhibitors, including Zn^2+^, small-molecule inhibitors, and venom-derived peptides, have been identified to inhibit Hv1 function (Fig. 5).

### 3.1. Zn^2+^

The inhibitory effect of Zn^2+^ is dependent on external pH and remains unaffected by internal pH, suggesting competitive interaction between protons and Zn^2+^ at external binding sites on Hv1 [87]. Two histidine residues (H140 and H193 in human Hv1) were identified to be critical for Zn^2+^-mediated inhibition [9, 10, 88–90], and Zn^2+^ was shown to inhibit Hv1 currents by binding to different closed states of the channel [90].

Zn^2+^ has been widely used for the role of Hv1 proton channel in neutrophils [32,34,91,92]. Zn^2+^ inhibited proton channels and significantly decreased intracellular pH in human neutrophils [32]. The effects of Zn^2+^ on intracellular pH were highly concentration dependent. While 30 μM Zn^2+^ caused only minor changes, maximal acidification was observed at 100 μM, with no further increase in acidification at 300 μM or 1 mM [32]. Moreover, neutrophil phagocytosis was reduced by 300 μM Zn^2+^ [32]. Other studies showed that inhibiting proton channels with Zn^2+^ resulted in profound acidification to levels and inhibition of ROS [91,92].

In human and mouse eosinophils, Zn^2+^ has been shown to inhibit Hv1 proton currents and production of ROS [50,51,53,93]. It is noted that the inhibition of eosinophil Hv1 required a much higher level of Zn^2+^ compared to neutrophil Hv1 [50], indicating differential regulation of Hv1 function in different immune cells. Moreover, Hv1-mediated proton currents could be abolished by 50 μM Zn^2+^ in mouse dendritic cells [82]. In human monocytes, 100 μM Zn^2+^ greatly reduced Hv1 proton currents and H_2_O_2_ production [56,94]. Treatment of 100 μM Zn^2+^ significantly reduced superoxide production and phagosomal pH in macrophages [62,95].

### 3.2. Small molecule inhibitors

#### 3.2.1. Guanidine derivatives

**Fig. 6.**
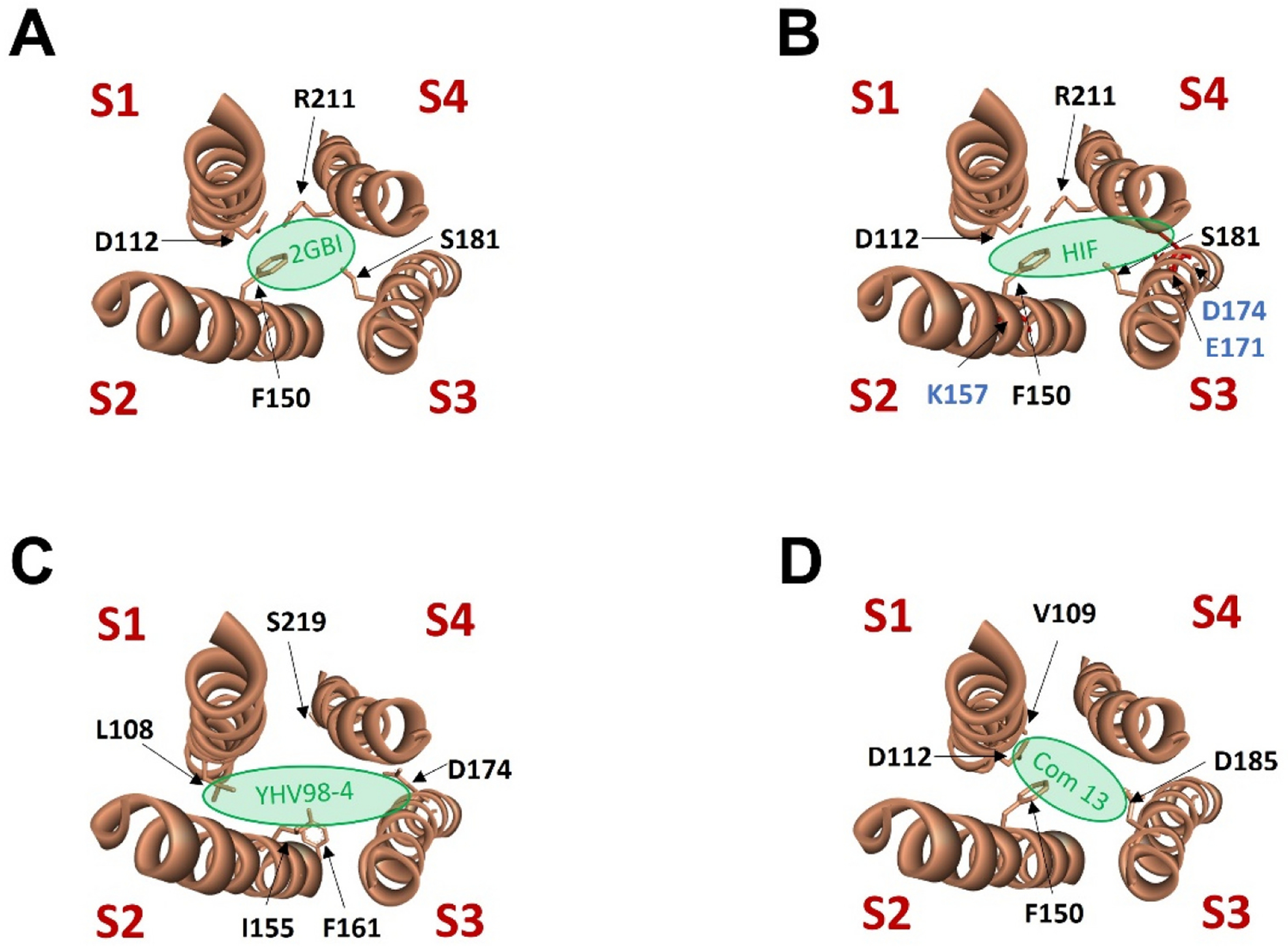
Binding sites of small-molecule inhibitors in human Hv1 channel, viewed from the extracellular side of the membrane. (A) Four residues (D112, F150, S181, and R211) form the binding sites of 2GBI. (B) HIF has two binding sites: the binding site 1 in the open channel overlaps the ones of 2GBI, and the binding site 2 in the closed channel involved in residues K157, E171, D174, and R211. (C) Some proposed binding sites of YHV98–4. (D) Proposed binding pocket of Compound 13.

Guanidine derivative 2-Guanidinobenzimidazole (2GBI) was identified to act as open channel blocker on human Hv1 channel [96,97] (Fig. 5B). The binding sites for this blocker are located at an intracellular vestibule surrounded by residues D112, F150, S181, and R211 of the human Hv1 channel [97] (Fig. 6A). A modified version of 2GBI (5-chloro-2GBI, or ClGBI) significantly increases the binding affinity of the channel and can reach the binding site on Hv1 from the extracellular side of the membrane [97].

ClGBI-mediated inhibition of Hv1 was shown to modify the viability of human polarized macrophages in a polarization-dependent manner [98]. ClGBI dose-dependently compromises the viability of polarized macrophages, and 50 μM ClGBI significantly reduces the viability of human THP-1-derived polarized macrophages, with M1 macrophages being the most sensitive and M2 macrophages the least sensitive. ClGBI-induced Hv1 inhibition leads to a pH dysregulation throughout the cell involving cytoplasmic and lysosomal compartments, which results in ceramide overproduction in polarized macrophages, and eventually the compromised viability of cells [98]. In myeloid-derived suppressor cells (MDSCs), inhibition of Hv1 by ClGBI resulted in intracellular acidification and decreased levels of ROS [84,85]. Similar results have been found in T cells, and ClGBI-mediated Hv1 inhibition induces acidification of leukemic Jurkat T cells promoting apoptosis [28]. Moreover, treatment of ClGBI reduced the inflammatory activation profile of microglia, and Hv1 inhibition by ClGBI promotes an anti-inflammatory activation phenotype in these cells [99]. Another study showed that 2GBI regulated efferocytosis of macrophages in human SLE [61]. Taken together, these findings suggested that Hv1 inhibitors show therapeutic promise in human pathological conditions such as neuroinflammation and malignant diseases. In addition to immune cells, 2GBI and ClGBI have been found to inhibit Hv1 channel and modify its function in cancer cell [100–102], heart cell [103], and sperm [104–106].

Another guanidine derivative, Hv1 Inhibitor Flexible (HIF), has been recently characterized to block Hv1 channel in the closed and open conformation [107,108] (Fig. 6B). HIF blocks ROS production in human and murine neutrophils infected with *Pseudomonas aeruginosa* [109]. Given that bacterial keratitis is a major cause of corneal disease where neutrophils contribute to stromal matrix degradation, targeting Hv1 with inhibitors such as HIF may help reduce inflammation and fibrosis associated with infection [109].

#### 3.2.2. YHV98-4

Using computer simulations and virtual screening of a chemical library, YHV98–4 was identified to bind to an intermediate conformational state of the Hv1 channel, a transition state between the resting and activated states [110,111]. The binding sites of YHV98–4 are proposed to be located at the intracellular side of the channel (Fig. 6C). 20 μM YHV98–4 could inhibit Hv1-mediated currents in mouse dorsal root ganglion (DRG) neurons. Treatment of YHV98–4 in mice attenuates inflammatory pain via inhibition of Hv1 and ROS production in DRG neurons and reduces morphine-induced adverse effects [110].

#### 3.2.3. Compound13

Using a structure-based approach, a new Hv1 inhibitor named Compound 13 was identified to block Hv1 proton current [112]. The effects of Compound 13 on the proliferation of MDA-MB-231 breast cancer cell line and THP-1 monocyte cell line were investigated, and Compound 13 inhibited growth with an IC_50_ value of 9.0 and 8.1 μM, respectively. The docking predicts that Compound 13 binds the core of the channel (Fig. 6D). These discoveries demonstrated the anticancer potential of the identified new Hv1 inhibitors [112].

#### 3.2.4. PNX52429 and PNX61442

One study applied cell-based High Throughput Screening (HTS) to identify two potential Hv1 inhibitors, PNX52429 and PNX61442 [113]. The binding sites of PNX52429 and PNX61442 have not been identified yet. Electrophysiological experiments showed that 50 μM PNX52429 inhibited around 50 % voltage-gated proton currents in mouse Ra2 microglial cell line, and 50 μM PNX61442 inhibited around 60 % voltage-gated proton currents in human eosinophils [113]. These findings suggested that PNX52429 and PNX61442 might serve as valuable tools for exploring the therapeutic potential of Hv1 inhibition in treating tumors and inflammatory diseases.

#### 3.2.5. Other drugs

Several neurological drugs have been reported to inhibit Hv1 channels in microglia [114–121]. Local anaesthetics such as lidocaine and bupivacaine were shown to inhibit voltage-gated proton currents in rat GMI-R1 microglia cell line [114]. In mouse BV2 microglial cell line, antidepressants, antitussive drugs, antipsychotics, and antagonists of histamine H1 receptors were reported to inhibit proton currents to some extent [115–121].

### 3.3. Peptide inhibitors

#### 3.3.1. C6

Using a phage-display library of variants based on the inhibitor cysteine knot scaffold, Corza6 (C6) was identified and characterized as a potent and specific inhibitor of human Hv1 [122]. C6 is composed of residues present in three natural parental spider toxins, including ω-ACTX-Hv1b, JZTX21, and Hm2. The binding sites of C6 are located at the external S3-S4 Loop of the human Hv1 channel [123]. And treatment of 1 μM C6 significantly inhibited ROS production in human neutrophils during the respiratory burst [122]. Recent studies showed that intravenous administration of C6 mitigates bacterial lipopolysaccharide (LPS)-induced acute lung injury (ALI) in mice by reducing neutrophil infiltration, ROS production, and proinflammatory cytokine levels in bronchoalveolar lavage fluid [124]. The findings identify Hv1 as a key regulator of ALI and support the potential of C6 as a therapeutic agent for lung injury.

#### 3.3.2. AGAP

Scorpion toxin AGAP (anti-tumour analgesic peptide) was found to inhibit Hv1 proton channel. One AGAP mutant AGAP/W38F blocks human Hv1 channel activation by trapping the S4 transmembrane domain of the channel in a deactivated state [125]. AGAP/W38F at 10 μM can abolish human Hv1 currents expressed in HEK293T cells. Mutation analysis revealed that the binding pockets for AGAP/W38F and Zn^2+^ on the Hv1 channel partially overlapped, with His140 and His193 identified as shared binding sites. Electrophysiological data and molecular dynamics simulations indicated that AGAP/W38F inhibits the Hv1 channel through a Zn^2+^-like long-range conformational coupling mechanism [125].

#### 3.3.3. HaTX

Tarantula toxin hanatoxin (HaTX) was shown to target the voltage sensor of voltage-gated ion channels and alter channel gating to inhibit ionic currents [126]. Extracellular application of 4 μM HaTX produced robust inhibition of voltage-gated Hv1 proton channels expressed in *Xenopus* oocytes. And HaTX shifts the activation of the Hv1 channel to more positive voltages, indicating that the treatment of HaTX can stabilize the closed state of the Hv1 channel [126].

## 4. Conclusion and perspective

The voltage-gated proton channel Hv1 plays a key role in regulating immune cell function by maintaining pH homeostasis, supporting ROS production, and modulating signaling pathways essential for innate and adaptive immune responses. Its expression in immune cells, including neutrophils, macrophages, eosinophils, B cells, and T cells, highlights its fundamental contribution to host defense, inflammation, and immune regulation. Dysregulation of Hv1 activity has been implicated in a variety of pathological conditions (Table 1), including autoimmune diseases [5], chronic inflammation [71,127,128], and cancer [101,102,129], making it a promising therapeutic target.

Despite the critical importance of Hv1, the field remains in an early stage with respect to pharmacological modulation. While classical inhibitors like Zn^2+^ have long been used to study Hv1 function, they lack specificity and are unsuitable for clinical applications. Recent efforts have led to the discovery of small-molecule inhibitors and venom-derived peptides with greater selectivity and efficacy. These pharmacological agents have demonstrated therapeutic potential in preclinical models of inflammatory diseases, acute lung injury, neuropathic pain, and cancer, providing proof-of-concept for Hv1-targeted interventions.

Future research should focus on several key areas. First, further elucidation of the structural basis of Hv1 gating and inhibition is essential. High-resolution structural studies, including cryo-electron microscopy and molecular dynamics simulations, are needed to define conformational changes during channel activation and inhibitor binding [130,131]. Such insights will enable rational drug design strategies aimed at targeting specific channel states or allosteric sites, thus improving the specificity and potency of Hv1-targeted compounds. Second, more comprehensive studies are required to understand the broader physiological and pathological roles of Hv1 across diverse immune contexts. Investigating the function of Hv1 not only in classical phagocytes like neutrophils and macrophages but also in adaptive immune cells such as T and B lymphocytes, dendritic cells, and tissue-resident immune cells will provide more views into its immunological impact. Furthermore, exploring Hv1 interaction with other proteins and intracellular signaling pathways may reveal novel mechanisms by which Hv1 modulates immune responses, inflammation, and tissue repair. Finally, translation of Hv1-targeted therapies into clinical practice will require careful evaluation of safety profiles. Given that Hv1 is integral to immune cell function, particularly in ROS production and microbial killing, inhibition of the channel should avoid compromising host defense or inducing immunosuppression. Preclinical studies should thoroughly evaluate immunological consequences, dosing strategies, and long-term effects.

Overall, advancing these research directions will not only deepen our understanding of the role of Hv1 in immune cells but also open new avenues for the treatment of Hv1-mediated immune and inflammatory disorders.

## Figures and Tables

**Table 1:** Hv1 and immune cell diseases.

Immune cell	Disease	The contribution of Hv1	Reference
B cell	B-cell malignancies	A key modulator of BCR signaling underlying the development of mantle cell lymphoma (MCL)	[21–23]
T cell	T-cell leukemia	Hv1 activity might prevent acidification of leukemic Jurkat T cells and protect against cancer cell death by apoptosis.	[28]
Neutrophil	Pseudomonas aeruginosa corneal infection	A critical role in sustaining ROS production by neutrophils to kill pathogenic P. aeruginosa in blinding corneal infection.	[109]
	Acute lung injury	A key regulator of acute lung injury.	[124]
Eosinophil	Chronic Granulomatous Disease	Regulates activation of eosinophil.	[52]
Monocyte	Staphylococcus aureus infection	Hv1 mediates LukAB toxins binding and cytotoxicity and promotes Staphylococcus aureus pathogenesis.	[58]
Macrophage	Autoimmune disease SLE	Lower Hv1-dependent acidification is associated with defective efferocytosis of macrophages.	[61]
Microglia	Ischemic stroke	Enhances brain damage from ischemic stroke.	[64]
	Spinal cord injury	Contributes to neuronal injury and motor deficits.	[68]
MDSC	Immunosuppressive disease	Responsible for ROS-mediated immunosuppression in Myeloid-derived suppressor cell (MDSC).	[84–86]

## References

[1] Medina KL, Overview of the immune system, Handb. Clin. Neurol 133 (2016) 61–76. 27112671 10.1016/B978-0-444-63432-0.00004-9

[2] Justiz Vaillant AA, Sabir S, Jan A Physiology, Immune Response. In. StatPearls. Treasure Island (FL); 2025. 30969623

[3] Sender R, Weiss Y, Navon Y, Milo I, Azulay N, Keren L, Fuchs S, Ben-Zvi D, Noor E, Milo R, The total mass, number, and distribution of immune cells in the human body, Proc. Natl. Acad. Sci. USA 120 (44) (2023) e2308511120. 37871201 10.1073/pnas.2308511120 PMC10623016

[4] Capasso M, DeCoursey TE, Dyer MJ, pH regulation and beyond: unanticipated functions for the voltage-gated proton channel, HVCN1, Trends Cell Biol. 21 (1) (2011) 20–28. 20961760 10.1016/j.tcb.2010.09.006 PMC3014425

[5] Capasso M, Regulation of immune responses by proton channels, Immunology 143 (2) (2014) 131–137. 24890927 10.1111/imm.12326 PMC4172129

[6] Capasso M, Proton channels in non-phagocytic cells of the immune system, Wiley Inter. Rev. Membr. Transp. Signal 2 (2) (2013) 65–73. 10.1002/wmts.78 PMC3659412 23710424

[7] Yan L, Zhang L, Ogunniyi K, Hong L, Ion Channels in the Immune Response of Asthma, J. Respir. Biol. Transl. Med 1 (4) (2024). 10.70322/jrbtm.2024.10019 PMC11633837 39664985

[8] Wu X, Singla S, Liu JJ, Hong L, The role of macrophage ion channels in the progression of atherosclerosis, Front Immunol. 14 (2023) 1225178. 37588590 10.3389/fimmu.2023.1225178 PMC10425548

[9] Ramsey IS, Moran MM, Chong JA, Clapham DE, A voltage-gated proton-selective channel lacking the pore domain, Nature 440 (7088) (2006) 1213–1216. 16554753 10.1038/nature04700 PMC4084761

[10] Takeshita K, Sakata S, Yamashita E, Fujiwara Y, Kawanabe A, Kurokawa T, Okochi Y, Matsuda M, Narita H, Okamura Y, Nakagawa A, X-ray crystal structure of voltage-gated proton channel, Nat. Struct. Mol. Biol 21 (4) (2014) 352–357. 24584463 10.1038/nsmb.2783

[11] Bayrhuber M, Maslennikov I, Kwiatkowski W, Sobol A, Wierschem C, Eichmann C, Frey L, Riek R, Nuclear Magnetic Resonance Solution Structure and Functional Behavior of the Human Proton Channel, Biochemistry 58 (39) (2019) 4017–4027. 31365236 10.1021/acs.biochem.9b00471

[12] Sasaki M, Takagi M, Okamura Y, A voltage sensor-domain protein is a voltage-gated proton channel, Science 312 (5773) (2006) 589–592. 16556803 10.1126/science.1122352

[13] Hong L, Tombola F, Allostery: A lipid two-step, Nat. Chem. Biol 12 (4) (2016) 202–203. 26878551 10.1038/nchembio.2037 PMC5940328

[14] DeCoursey TE, Voltage-gated proton channels: molecular biology, physiology, and pathophysiology of the H(V) family, Physiol. Rev 93 (2) (2013) 599–652. 23589829 10.1152/physrev.00011.2012 PMC3677779

[15] Wu X, Li Y, Maienschein-Cline M, Feferman L, Wu L, Hong L, RNA-Seq Analyses Reveal Roles of the HVCN1 Proton Channel in Cardiac pH Homeostasis, Front Cell Dev. Biol 10 (2022) 860502. 35372367 10.3389/fcell.2022.860502 PMC8967321

[16] Wu X, Zhang L, Hong L, The role of Phe150 in human voltage-gated proton channel, iScience 25 (11) (2022) 105420. 36388967 10.1016/j.isci.2022.105420 PMC9646954

[17] Zhang L, Wu X, Cao X, Rao K, Hong L, Trp207 regulation of voltage-dependent activation of human H(v)1 proton channel, J. Biol. Chem 300 (3) (2024) 105674. 38272234 10.1016/j.jbc.2024.105674 PMC10875263

[18] Checa J, Aran JM, Reactive Oxygen Species: Drivers of Physiological and Pathological Processes, J. Inflamm. Res 13 (2020) 1057–1073. 33293849 10.2147/JIR.S275595 PMC7719303

[19] Capasso M, Bhamrah MK, Henley T, Boyd RS, Langlais C, Cain K, Dinsdale D, Pulford K, Khan M, Musset B, Cherny VV, Morgan D, Gascoyne RD, Vigorito E, DeCoursey TE, MacLennan IC, Dyer MJ, HVCN1 modulates BCR signal strength via regulation of BCR-dependent generation of reactive oxygen species, Nat. Immunol 11 (3) (2010) 265–272. 20139987 10.1038/ni.1843 PMC3030552

[20] Reth M, Dick TP, Voltage control for B cell activation, Nat. Immunol 11 (3) (2010) 191–192. 20157299 10.1038/ni0310-191

[21] Boyd RS, Dyer MJ, Cain K, Proteomic analysis of B-cell malignancies, J. Proteom 73 (10) (2010) 1804–1822. 10.1016/j.jprot.2010.03.010 20346427

[22] Hondares E, Brown MA, Musset B, Morgan D, Cherny VV, Taubert C, Bhamrah MK, Coe D, Marelli-Berg F, Gribben JG, Dyer MJ, DeCoursey TE, Capasso M, Enhanced activation of an amino-terminally truncated isoform of the voltage-gated proton channel HVCN1 enriched in malignant B cells, Proc. Natl. Acad. Sci. USA 111 (50) (2014) 18078–18083. 25425665 10.1073/pnas.1411390111 PMC4273330

[23] Krysiak K, Gomez F, White BS, Matlock M, Miller CA, Trani L, Fronick CC, Fulton RS, Kreisel F, Cashen AF, Carson KR, Berrien-Elliott MM, Bartlett NL, Griffith M, Griffith OL, Fehniger TA, Recurrent somatic mutations affecting B-cell receptor signaling pathway genes in follicular lymphoma, Blood 129 (4) (2017) 473–483. 28064239 10.1182/blood-2016-07-729954 PMC5270390

[24] Sasaki M, Tojo A, Okochi Y, Miyawaki N, Kamimura D, Yamaguchi A, Murakami M, Okamura Y, Autoimmune disorder phenotypes in Hvcn1-deficient mice, Biochem J. 450 (2) (2013) 295–301. 23231444 10.1042/BJ20121188

[25] Kasic T, Colombo P, Soldani C, Wang CM, Miranda E, Roncalli M, Bronte V, Viola A, Modulation of human T-cell functions by reactive nitrogen species, Eur. J. Immunol 41 (7) (2011) 1843–1849. 21480210 10.1002/eji.201040868

[26] Coe D, Poobalasingam T, Fu H, Bonacina F, Wang G, Morales V, Moregola A, Mitro N, Cheung KC, Ward EJ, Nadkarni S, Aksentijevic D, Bianchi K, Norata GD, Capasso M, Marelli-Berg FM, Loss of voltage-gated hydrogen channel 1 expression reveals heterogeneous metabolic adaptation to intracellular acidification by T cells, JCI Insight 7 (10) (2022). 10.1172/jci.insight.147814 PMC9220931 35472029

[27] Schilling T, Gratopp A, DeCoursey TE, Eder C, Voltage-activated proton currents in human lymphocytes, J. Physiol 545 (1) (2002) 93–105. 12433952 10.1113/jphysiol.2002.028878 PMC2290658

[28] Asuaje A, Smaldini P, Martin P, Enrique N, Orlowski A, Aiello EA, Gonzalez Leon C, Docena G, Milesi V, The inhibition of voltage-gated H(+) channel (HVCN1) induces acidification of leukemic Jurkat T cells promoting cell death by apoptosis, Pflug. Arch 469 (2) (2017) 251–261. 10.1007/s00424-016-1928-0 28013412

[29] Asuaje A, Martin P, Enrique N, Zegarra LAD, Smaldini P, Docena G, Milesi V, Diphenhydramine inhibits voltage-gated proton channels (Hv1) and induces acidification in leukemic Jurkat T cells- New insights into the pro-apoptotic effects of antihistaminic drugs, Channels (Austin) 12 (1) (2018) 58–64. 28514187 10.1080/19336950.2017.1331799 PMC5972794

[30] Navarro F, Casares N, Martin-Otal C Lasarte-Cia A, Gorraiz M, Sarrion P, Llopiz D, Reparaz D, Varo N, Rodriguez-Madoz JR, Prosper F, Hervas-Stubbs S, Lozano T, Lasarte JJ, Overcoming T cell dysfunction in acidic pH to enhance adoptive T cell transfer immunotherapy, Oncoimmunology 11 (1) (2022) 2070337. 35529677 10.1080/2162402X.2022.2070337 PMC9067511

[31] Okochi Y, Okamura Y, Regulation of Neutrophil Functions by Hv1/VSOP Voltage-Gated Proton Channels, Int J. Mol. Sci 22 (5) (2021). 10.3390/ijms22052620 PMC7961965 33807711

[32] Morgan D, Capasso M, Musset B, Cherny VV, Rios E, Dyer MJ, DeCoursey TE, Voltage-gated proton channels maintain pH in human neutrophils during phagocytosis, Proc. Natl. Acad. Sci. USA 106 (42) (2009) 18022–18027. 19805063 10.1073/pnas.0905565106 PMC2764923

[33] DeCoursey TE, Cherny VV, Zhou W, Thomas LL, Simultaneous activation of NADPH oxidase-related proton and electron currents in human neutrophils, Proc. Natl. Acad. Sci. USA 97 (12) (2000) 6885–6889. 10823889 10.1073/pnas.100047297 PMC18770

[34] Ratanayotha A, Kawai T, Higashijima SI, Okamura Y, Molecular and functional characterization of the voltage-gated proton channel in zebrafish neutrophils, Physiol. Rep 5 (15) (2017). 10.14814/phy2.13345 PMC5555884 28774948

[35] Okochi Y, Umemoto E, Okamura Y, Hv1/VSOP regulates neutrophil directional migration and ERK activity by tuning ROS production, J. Leukoc. Biol 107 (5) (2020) 819–831. 32303121 10.1002/JLB.2A0320-110RR

[36] Okochi Y, Aratani Y, Adissu HA, Miyawaki N, Sasaki M, Suzuki K, Okamura Y, The voltage-gated proton channel Hv1/VSOP inhibits neutrophil granule release, J. Leukoc. Biol 99 (1) (2016) 7–19. 25990245 10.1189/jlb.3HI0814-393R

[37] El Chemaly A, Nunes P, Jimaja W, Castelbou C, Demaurex N, Hv1 proton channels differentially regulate the pH of neutrophil and macrophage phagosomes by sustaining the production of phagosomal ROS that inhibit the delivery of vacuolar ATPases, J. Leukoc. Biol 95 (5) (2014) 827–839. 24415791 10.1189/jlb.0513251

[38] El Chemaly A, Okochi Y, Sasaki M, Arnaudeau S, Okamura Y, Demaurex N, VSOP/Hv1 proton channels sustain calcium entry, neutrophil migration, and superoxide production by limiting cell depolarization and acidification, J. Exp. Med 207 (1) (2010) 129–139. 20026664 10.1084/jem.20091837 PMC2812533

[39] Kovacs I, Horvath M, Kovacs T, Somogyi K, Tretter L, Geiszt M, Petheo GL, Comparison of proton channel, phagocyte oxidase, and respiratory burst levels between human eosinophil and neutrophil granulocytes, Free Radic. Res 48 (10) (2014) 1190–1199. 24985354 10.3109/10715762.2014.938234

[40] Demaurex N, Schrenzel J, Jaconi ME, Lew DP, Krause KH, Proton channels, plasma membrane potential, and respiratory burst in human neutrophils, Eur. J. Haematol 51 (5) (1993) 309–312. 8282093 10.1111/j.1600-0609.1993.tb01613.x

[41] DeCoursey TE, Cherny VV, Potential, pH, and arachidonate gate hydrogen ion currents in human neutrophils, Biophys. J 65 (4) (1993) 1590–1598. 7506066 10.1016/S0006-3495(93)81198-6 PMC1225885

[42] Ratanayotha A, Kawai T, Okamura Y, Real-time functional analysis of Hv1 channel in neutrophils: a new approach from zebrafish model, Am. J. Physiol. Regul. Integr. Comp. Physiol 316 (6) (2019) R819–R831. 30943046 10.1152/ajpregu.00326.2018

[43] Okochi Y, Sasaki M, Iwasaki H, Okamura Y, Voltage-gated proton channel is expressed on phagosomes, Biochem Biophys. Res Commun 382 (2) (2009) 274–279. 19285483 10.1016/j.bbrc.2009.03.036

[44] El Chemaly A, Demaurex N, Do Hv1 proton channels regulate the ionic and redox homeostasis of phagosomes? Mol. Cell Endocrinol 353 (1-2) (2012) 82–87. 22056415 10.1016/j.mce.2011.10.005

[45] Gordienko DV, Tare M, Parveen S, Fenech CJ, Robinson C, Bolton TB, Voltage-activated proton current in eosinophils from human blood, J. Physiol 496 (Pt 2) (1996) 299–316. 8910217 10.1113/jphysiol.1996.sp021686 PMC1160878

[46] Schrenzel J, Lew DP, Krause KH, Proton currents in human eosinophils, Am. J. Physiol 271 (6 Pt 1) (1996) C1861–C1871. 8997186 10.1152/ajpcell.1996.271.6.C1861

[47] Cherny VV, Henderson LM, Xu W, Thomas LL, DeCoursey TE, Activation of NADPH oxidase-related proton and electron currents in human eosinophils by arachidonic acid, J. Physiol 535 (Pt 3) (2001) 783–794. 11559775 10.1111/j.1469-7793.2001.00783.x PMC2278825

[48] Cherny VV, Murphy R, Sokolov V, Levis RA, DeCoursey TE, Properties of single voltage-gated proton channels in human eosinophils estimated by noise analysis and by direct measurement, J. Gen. Physiol 121 (6) (2003) 615–628. 12771195 10.1085/jgp.200308813 PMC2217352

[49] Morgan D, Cherny VV, Finnegan A, Bollinger J, Gelb MH, DeCoursey TE, Sustained activation of proton channels and NADPH oxidase in human eosinophils and murine granulocytes requires PKC but not cPLA2 alpha activity, J. Physiol 579 (Pt 2) (2007) 327–344. 17185330 10.1113/jphysiol.2006.124248 PMC2075394

[50] DeCoursey TE, Morgan D, Cherny VV, The voltage dependence of NADPH oxidase reveals why phagocytes need proton channels, Nature 422 (6931) (2003) 531–534. 12673252 10.1038/nature01523

[51] DeCoursey TE, Cherny VV, DeCoursey AG, Xu W, Thomas LL, Interactions between NADPH oxidase-related proton and electron currents in human eosinophils, J. Physiol 535 (Pt 3) (2001) 767–781. 11559774 10.1111/j.1469-7793.2001.00767.x PMC2278831

[52] Banfi B, Schrenzel J, Nusse O, Lew DP, Ligeti E, Krause KH, Demaurex N, A novel H(+) conductance in eosinophils: unique characteristics and absence in chronic granulomatous disease, J. Exp. Med 190 (2) (1999) 183–194. 10432282 10.1084/jem.190.2.183 PMC2195580

[53] Zhu X, Mose E, Zimmermann N, Proton channel HVCN1 is required for effector functions of mouse eosinophils, BMC Immunol. 14 (2013) 24. 23705768 10.1186/1471-2172-14-24 PMC3668235

[54] Musset B, Morgan D, Cherny VV, MacGlashan DW Jr., Thomas LL, Rios E, DeCoursey TE, A pH-stabilizing role of voltage-gated proton channels in IgE-mediated activation of human basophils, Proc. Natl. Acad. Sci. USA 105 (31) (2008) 11020–11025. 18664579 10.1073/pnas.0800886105 PMC2504794

[55] Kuno M, Kawawaki J, Nakamura F, A highly temperature-sensitive proton current in mouse bone marrow-derived mast cells, J. Gen. Physiol 109 (6) (1997) 731–740. 9222899 10.1085/jgp.109.6.731 PMC2217037

[56] DeCoursey TE, Cherny VV, Voltage-activated proton currents in human THP-1 monocytes, J. Membr. Biol 152 (2) (1996) 131–140. 9139124 10.1007/s002329900092

[57] DeCoursey TE, Kim SY, Silver MR, Quandt FN Ion channel expression in PMA-differentiated human THP-1 macrophages, J. Membr. Biol 152 (2) (1996) 141–157. 9139125 10.1007/s002329900093

[58] Perelman SS, James DBA, Boguslawski KM, Nelson CW, Ilmain JK, Zwack EE, Prescott RA, Mohamed A, Tam K, Chan R, Narechania A, Pawline MB, Vozhilla N, Moustafa AM, Kim SY, Dittmann M, Ekiert DC, Bhabha G, Shopsin B, Planet PJ, Koralov SB, Torres VJ, Genetic variation of staphylococcal LukAB toxin determines receptor tropism, Nat. Microbiol 6 (6) (2021) 731–745. 33875847 10.1038/s41564-021-00890-3 PMC8597016

[59] Kapus A, Romanek R, Qu AY, Rotstein OD, Grinstein S, A pH-sensitive and voltage-dependent proton conductance in the plasma membrane of macrophages, J. Gen. Physiol 102 (4) (1993) 729–760. 8270911 10.1085/jgp.102.4.729 PMC2229171

[60] Nelson DJ, Jow B, Jow F, Whole-cell currents in macrophages: I. Human monocyte-derived macrophages, J. Membr. Biol 117 (1) (1990) 29–44. 2402007 10.1007/BF01871563

[61] Geng L, Zhao J, Deng Y, Molano I, Xu X, Xu L, Ruiz P, Li Q, Feng X, Zhang M, Tan W, Kamen DL, Bae SC, Gilkeson GS, Sun L, Tsao BP, Human SLE variant NCF1-R90H promotes kidney damage and murine lupus through enhanced Tfh2 responses induced by defective efferocytosis of macrophages, Ann. Rheum. Dis 81 (2) (2022) 255–267. 34556485 10.1136/annrheumdis-2021-220793

[62] Jin C, Sun J, Stilphen CA, Smith SM, Ocasio H, Bermingham B, Darji S, Guha A, Patel R, Geurts AM, Jacob HJ, Lambert NA, O’Connor PM, HV1 acts as a sodium sensor and promotes superoxide production in medullary thick ascending limb of Dahl salt-sensitive rats, Hypertension 64 (3) (2014) 541–550. 24935944 10.1161/HYPERTENSIONAHA.114.03549 PMC4324599

[63] Patel B, Zheleznova NN, Ray SC, Sun J, Cowley AW, O. Jr, ’Connor PM Voltage gated proton channels modulate mitochondrial reactive oxygen species production by complex I in renal medullary thick ascending limb, Redox Biol. 27 (2019) 101191. 31060879 10.1016/j.redox.2019.101191 PMC6859587

[64] Wu LJ, Wu G, Sharif Akhavan, Baker MR, Jia A, Fahey Y, Luo FH, Feener HR, Clapham EP, DE. The voltage-gated proton channel Hv1 enhances brain damage from ischemic stroke, Nat. Neurosci 15 (4) (2012) 565–573. 22388960 10.1038/nn.3059 PMC3314139

[65] Wu LJ, Voltage-gated proton channel HV1 in microglia, Neuroscientist 20 (6) (2014) 599–609. 24463247 10.1177/1073858413519864

[66] Wu LJ, Microglial voltage-gated proton channel Hv1 in ischemic stroke, Transl. Stroke Res 5 (1) (2014) 99–108. 24323712 10.1007/s12975-013-0289-7

[67] Kawai T, Tatsumi S, Kihara S, Sakimura K, Okamura Y, Mechanistic insight into the suppression of microglial ROS production by voltage-gated proton channels (VSOP/Hv1), Channels 12 (1) (2018) 1–8. 28961043 10.1080/19336950.2017.1385684 PMC5972804

[68] Murugan M, Zheng J, Wu G, Mogilevsky R, Zheng X, Hu P, Wu J, Wu LJ, The voltage-gated proton channel Hv1 contributes to neuronal injury and motor deficits in a mouse model of spinal cord injury, Mol. Brain 13 (1) (2020) 143. 33081841 10.1186/s13041-020-00682-6 PMC7574559

[69] Li W, Ward R, Dong G, Ergul A, O’Connor P, Neurovascular protection in voltage-gated proton channel Hv1 knock-out rats after ischemic stroke: interaction with Na(+) /H(+) exchanger-1 antagonism, Physiol. Rep 7 (13) (2019) e14142. 31250553 10.14814/phy2.14142 PMC6597793

[70] Li X, Yu Z, Zong W, Chen P, Li J, Wang M, Ding F, Xie M, Wang W, Luo X, Deficiency of the microglial Hv1 proton channel attenuates neuronal pyroptosis and inhibits inflammatory reaction after spinal cord injury, J. Neuroinflamm 17 (1) (2020) 263. 10.1186/s12974-020-01942-x PMC7487532 32891159

[71] Li Y, Ritzel RM, He J, Cao T, Sabirzhanov B, Li H, Liu S, Wu LJ, Wu J, The voltage-gated proton channel Hv1 plays a detrimental role in contusion spinal cord injury via extracellular acidosis-mediated neuroinflammation, Brain Behav. Immun 91 (2021) 267–283. 33039662 10.1016/j.bbi.2020.10.005 PMC7749852

[72] Zheng J, Murugan M, Wang L, Wu LJ, Microglial voltage-gated proton channel Hv1 in spinal cord injury, Neural Regen. Res 17 (6) (2022) 1183–1189. 34782552 10.4103/1673-5374.327325 PMC8643068

[73] Tang Y, Wu X, Li J, Li Y, Xu X, Li G, Zhang P, Qin C, Wu LJ, Tang Z, Tian DS, The Emerging Role of Microglial Hv1 as a Target for Immunomodulation in Myelin Repair, Aging Dis. 15 (3) (2024) 1176–1203. 38029392 10.14336/AD.2023.1107 PMC11081154

[74] Tian DS, Li CY, Qin C, Murugan M, Wu LJ, Liu JL, Deficiency in the voltage-gated proton channel Hv1 increases M2 polarization of microglia and attenuates brain damage from photothrombotic ischemic stroke, J. Neurochem 139 (1) (2016) 96–105. 27470181 10.1111/jnc.13751 PMC5037018

[75] Marcoline FV, Ishida Y, Mindell JA, Nayak S, Grabe M, A mathematical model of osteoclast acidification during bone resorption, Bone 93 (2016) 167–180. 27650914 10.1016/j.bone.2016.09.007 PMC5077641

[76] Mori H, Sakai H, Morihata H, Yamano T, Kuno M, A voltage-gated H+ channel is a powerful mechanism for pH homeostasis in murine osteoclasts, Kobe J. Med. Sci 48 (3-4) (2002) 87–96. 12502906

[77] Sakai H, Li G, Hino Y, Moriura Y, Kawawaki J, Sawada M, Kuno M, Increases in intracellular pH facilitate endocytosis and decrease availability of voltage-gated proton channels in osteoclasts and microglia, J. Physiol 591 (23) (2013) 5851–5866. 24081153 10.1113/jphysiol.2013.263558 PMC3872757

[78] Mori H, Sakai H, Morihata H, Kawawaki J, Amano H, Yamano T, Kuno M, Regulatory mechanisms and physiological relevance of a voltage-gated H+ channel in murine osteoclasts: phorbol myristate acetate induces cell acidosis and the channel activation, J. Bone Min. Res 18 (11) (2003) 2069–2076. 10.1359/jbmr.2003.18.11.2069 14606521

[79] Kuno M, Li G, Moriura Y, Hino Y, Kawawaki J, Sakai H, Acid-inducible proton influx currents in the plasma membrane of murine osteoclast-like cells, Pflug. Arch 468 (5) (2016) 837–847. 10.1007/s00424-016-1796-7 26843093

[80] Li G, Miura K, Kuno M, Extracellular phosphates enhance activities of voltage-gated proton channels and production of reactive oxygen species in murine osteoclast-like cells, Pflug. Arch 469 (2) (2017) 279–292. 10.1007/s00424-016-1931-5 27999941

[81] Kawai T, Kayama K, Tatsumi S, Akter S, Miyawaki N, Okochi Y, Abe M, Sakimura K, Yamamoto H, Kihara S, Okamura Y, Regulation of hepatic oxidative stress by voltage-gated proton channels (Hv1/VSOP) in Kupffer cells and its potential relationship with glucose metabolism, FASEB J. 34 (12) (2020) 15805–15821. 33040408 10.1096/fj.202001056RRR

[82] Szteyn K, Yang W, Schmid E, Lang F, Shumilina E, H. Lipopolysaccharide-sensitive, current in dendritic cells, Am. J. Physiol. Cell Physiol 303 (2) (2012) C204–C212. 22572846 10.1152/ajpcell.00059.2012

[83] Montes-Cobos E, Huscher B, Engler JB, Woo MS, Binkle L, Bauer S, Kursawe N, Moles M, Friese MA, Ufer F, Voltage-gated proton channel Hv1 controls TLR9 activation in plasmacytoid dendritic cells, J. Immunol 205 (11) (2020) 3001–3010. 33127821 10.4049/jimmunol.2000404

[84] Alvear-Arias JJ, Carrillo C, Villar JP, Garcia-Betancourt R, Pena-Pichicoi A, Fernandez A, Fernandez M, Carmona EM, Pupo A, Neely A, Alvarez O, Garate J, Barajas-Martinez H, Larsson HP, Lopez-Rodriguez A, Latorre R, Gonzalez C, Expression of H(v)1 proton channels in myeloid-derived suppressor cells (MDSC) and its potential role in T cell regulation, Proc. Natl. Acad. Sci 119 (15) (2022) e2104453119. 35377790 10.1073/pnas.2104453119 PMC9169626

[85] Cozzolino M, Gyongyosi A, Korpos E, Gogolak P, Naseem MU, Kallai J, Lanyi A, Panyi G, The voltage-gated Hv1H(+) channel is expressed in tumor-infiltrating myeloid-derived suppressor cells, Int. J. Mol. Sci 24 (7) (2023). 10.3390/ijms24076216 PMC10094655 37047188

[86] Pena-Pichicoi A, Fernandez M, Navarro-Quezada N, Alvear-Arias JJ, Carrillo CA, Carmona EM, Garate J, Lopez-Rodriguez AM, Neely A, Hernandez-Ochoa EO, Gonzalez C, N-terminal region is responsible for mHv1 channel activity in MDSCs, Front Pharm. 14 (2023) 1265130. 10.3389/fphar.2023.1265130 PMC10616795 37915407

[87] Cherny VV, DeCoursey TE, pH-dependent inhibition of voltage-gated H(+) currents in rat alveolar epithelial cells by Zn(2+) and other divalent cations, J. Gen. Physiol 114 (6) (1999) 819–838. 10578017 10.1085/jgp.114.6.819 PMC2230650

[88] Iwaki M, Takeshita K, Kondo HX, Kinoshita K, Okamura Y, Takano Y, Nakagawa A, Kandori H, Zn(2+)-binding to the voltage-gated proton channel Hv1/VSOP, J. Phys. Chem. B 122 (39) (2018) 9076–9080. 30204443 10.1021/acs.jpcb.8b04890

[89] De La Rosa V, Bennett AL, Ramsey IS, Coupling between an electrostatic network and the Zn(2+) binding site modulates Hv1 activation, J. Gen. Physiol 150 (6) (2018) 863–881. 29743298 10.1085/jgp.201711822 PMC5987874

[90] Qiu F, Chamberlin A, Watkins BM, Ionescu A, Perez ME, Barro-Soria R, Gonzalez C, Noskov SY, Larsson HP, Molecular mechanism of Zn2+ inhibition of a voltage-gated proton channel, Proc. Natl. Acad. Sci 113 (40) (2016) E5962–E5971. 27647906 10.1073/pnas.1604082113 PMC5056077

[91] Levine AP, Duchen MR, de Villiers S, Rich PR, Segal AW, Alkalinity of neutrophil phagocytic vacuoles is modulated by HVCN1 and has consequences for myeloperoxidase activity, PLoS One 10 (4) (2015) e0125906. 25885273 10.1371/journal.pone.0125906 PMC4401748

[92] Droste A, Chaves G, Stein S, Trzmiel A, Schweizer M, Karl H, Musset B, Zinc accelerates respiratory burst termination in human PMN, Redox Biol. 47 (2021) 102133. 34562872 10.1016/j.redox.2021.102133 PMC8476447

[93] Petheo GL, Girardin NC, Goossens N, Molnar GZ, Demaurex N, Role of nucleotides and phosphoinositides in the stability of electron and proton currents associated with the phagocytic NADPH oxidase, Biochem J. 400 (3) (2006) 431–438. 16842239 10.1042/BJ20060578 PMC1698601

[94] Musset B, Cherny VV, DeCoursey TE, Strong glucose dependence of electron current in human monocytes, Am. J. Physiol. Cell Physiol 302 (1) (2012) C286–C295. 22012327 10.1152/ajpcell.00335.2011 PMC3328910

[95] Canton J, Khezri R, Glogauer M, Grinstein S, Contrasting phagosome pH regulation and maturation in human M1 and M2 macrophages, Mol. Biol. Cell 25 (21) (2014) 3330–3341. 25165138 10.1091/mbc.E14-05-0967 PMC4214780

[96] Hong L, Pathak MM, Kim IH, Ta D, Tombola F, Voltage-sensing domain of voltage-gated proton channel Hv1 shares mechanism of block with pore domains, Neuron 77 (2) (2013) 274–287. 23352164 10.1016/j.neuron.2012.11.013 PMC3559007

[97] Hong L, Kim IH, Tombola F, Molecular determinants of Hv1 proton channel inhibition by guanidine derivatives, Proc. Natl. Acad. Sci 111 (27) (2014) 9971–9976. 24912149 10.1073/pnas.1324012111 PMC4103315

[98] Kovacs T, Szabo Cs, Kothalawala B, Szekelyhidi RC, Nagy V, Varga P, Panyi Z, Zakany G, F. Inhibition of the H(V)1 voltage-gated proton channel compromises the viability of human polarized macrophages in a polarization- and ceramide-dependent manner, Front Immunol. 15 (2024) 1487578. 39742270 10.3389/fimmu.2024.1487578 PMC11685079

[99] Hernandez-Espinosa DR, Gale JR, Scrabis MG, Aizenman E, Microglial reprogramming by Hv1 antagonism protects neurons from inflammatory and glutamate toxicity, J. Neurochem 165 (1) (2023) 29–54. 36625847 10.1111/jnc.15760 PMC10106429

[100] Ventura C, Leon IE, Asuaje A, Martin P, Enrique N, Nunez M, Cocca C, Milesi V, Differential expression of the long and truncated Hv1 isoforms in breast-cancer cells, J. Cell Physiol (2020). 10.1002/jcp.29719 32324259

[101] Alvear-Arias JJ, Pena-Pichicoi A, Carrillo C, Fernandez M, Gonzalez T, Garate JA, Gonzalez C, Role of voltage-gated proton channel (Hv1) in cancer biology, Front Pharm 14 (2023) 1175702. 10.3389/fphar.2023.1175702 PMC10157179 37153807

[102] Fernandez A, Pupo A, Mena-Ulecia K, Gonzalez C, PharmacologicaL Modulation of Proton Channel Hv1 in cancer therapy: future perspectives, Mol. Pharm 90 (3) (2016) 385–402. 10.1124/mol.116.103804 27260771

[103] Ma J, Gao X, Li Y, DeCoursey TE, Shull GE, Wang HS, The HVCN1 voltage-gated proton channel contributes to pH regulation in canine ventricular myocytes, J. Physiol 600 (9) (2022) 2089–2103. 35244217 10.1113/JP282126 PMC9058222

[104] Yeste M, Llavanera M, Mateo-Otero Y, Catalan J, Bonet S, Pinart E, HVCN1 channels are relevant for the maintenance of sperm motility during in vitro capacitation of pig spermatozoa, Int. J. Mol. Sci 21 (9) (2020). 10.3390/ijms21093255 PMC7246839 32375375

[105] Delgado-Bermudez A, Mateo-Otero Y, Llavanera M, Bonet S, Yeste M, Pinart E, HVCN1 but not potassium channels are related to mammalian sperm cryotolerance, Int. J. Mol. Sci 22 (4) (2021). 10.3390/ijms22041646 PMC7914938 33562049

[106] Matamoros-Volante A, Trevino CL, Capacitation-associated alkalization in human sperm is differentially controlled at the subcellular level, J. Cell Sci (2020). 10.1242/jcs.238816 31932506

[107] Zhao C, Hong L, Riahi S, Lim VT, Tobias DJ, Tombola F, A novel Hv1 inhibitor reveals a new mechanism of inhibition of a voltage-sensing domain, J. Gen. Physiol 153 (9) (2021). 10.1085/jgp.202012833 PMC8263925 34228045

[108] Zhao C, Hong L, Galpin JD, Riahi S, Lim VT, Webster PD, Tobias DJ, Ahern CA, Tombola F, HIFs: New arginine mimic inhibitors of the Hv1 channel with improved VSD-ligand interactions, J. Gen. Physiol 153 (9) (2021). 10.1085/jgp.202012832 PMC8263924 34228044

[109] Rodriguez P, Abbondante S, Marshall M, Abdelmeseh J, Tombola F, Pearlman E, An essential role for the Hv1 voltage-gated proton channel in Pseudomonas aeruginosa corneal infection, bioRxiv (2024). 10.1093/jleuko/qiaf147 41121541

[110] Zhang Q, Ren Y, Mo Y, Guo P, Liao P, Luo Y, Mu J, Chen Z, Zhang Y, Li Y, Yang L, Liao D, Fu J, Shen J, Huang W, Xu X, Guo Y, Mei L, Zuo Y, Liu J, Yang H, Jiang R, Inhibiting Hv1 channel in peripheral sensory neurons attenuates chronic inflammatory pain and opioid side effects, Cell Res 32 (5) (2022) 461–476. 35115667 10.1038/s41422-022-00616-y PMC9061814

[111] Furutani K, Ji RR, Targeting Hv1 proton channel for pain control, Cell Res 32 (5) (2022) 419–420. 35365754 10.1038/s41422-022-00648-4 PMC9061828

[112] Piga M, Varga Z, Feher A, Papp F, Korpos E, Bangera KC, Frlan R, Ilas J, Dernovsek J, Tomasic T, Zidar N, Identification of a novel structural class of H (V)1 inhibitors by structure-based virtual screening, J. Chem. Inf. Model 64 (12) (2024) 4850–4862. 38850237 10.1021/acs.jcim.4c00240 PMC11200261

[113] El Chemaly A, Jaquet V, Cambet Y, Caillon A, Cherpin O, Balafa A, Krause KH, Demaurex N, Discovery and validation of new Hv1 proton channel inhibitors with onco-therapeutic potential, Biochim Biophys. Acta Mol. Cell Res 1870 (3) (2023) 119415. 36640925 10.1016/j.bbamcr.2022.119415

[114] Matsuura T, Mori T, Hasaka M, Kuno M, Kawawaki J, Nishikawa K, Narahashi T, Sawada M, Asada A, Inhibition of voltage-gated proton channels by local anaesthetics in GMI-R1 rat microglia, J. Physiol 590 (4) (2012) 827–844. 22183729 10.1113/jphysiol.2011.218149 PMC3381313

[115] Kim J, Song JH, Inhibitory effects of antihistamines, diphenhydramine and chlorpheniramine, on proton currents in BV2 microglial cells, Eur. J. Pharm 798 (2017) 122–128. 10.1016/j.ejphar.2017.01.032 28131782

[116] Shin H, Kim J, Song JH, Clozapine and olanzapine inhibit proton currents in BV2 microglial cells, Eur. J. Pharm 755 (2015) 74–79. 10.1016/j.ejphar.2015.03.003 25771455

[117] Song JH, Yeh JZ, Dextromethorphan inhibition of voltage-gated proton currents in BV2 microglial cells, Neurosci. Lett 516 (1) (2012) 94–98. 22487729 10.1016/j.neulet.2012.03.065

[118] Kim J, Song JH, Thioxanthenes, chlorprothixene and flupentixol inhibit proton currents in BV2 microglial cells, Eur. J. Pharm 779 (2016) 31–37. 10.1016/j.ejphar.2016.03.009 26945819

[119] Shin H, Song JH, Antipsychotics, chlorpromazine and haloperidol inhibit voltage-gated proton currents in BV2 microglial cells, Eur. J. Pharm 738 (2014) 256–262. 10.1016/j.ejphar.2014.05.049 24990667

[120] Jin S, Park M, Song JH, -)- Epigallocatechin-3-gallate inhibits voltage-gated proton currents in BV2 microglial cells, Eur. J. Pharm 698 (1-3) (2013) 154–160. 10.1016/j.ejphar.2012.11.036 23201067

[121] Song JH, Marszalec W, Kai L, Yeh JZ, Narahashi T, Antidepressants inhibit proton currents and tumor necrosis factor-alpha production in BV2 microglial cells, Brain Res. 1435 (2012) 15–23. 22177663 10.1016/j.brainres.2011.11.041

[122] Zhao R, Kennedy K, De Blas GA, Orta G, Pavarotti MA, Arias RJ, de la Vega-Beltran JL, Li Q, Dai H, Perozo E, Mayorga LS, Darszon A, Goldstein SAN, Role of human Hv1 channels in sperm capacitation and white blood cell respiratory burst established by a designed peptide inhibitor, Proc. Natl. Acad. Sci 115 (50) (2018) E11847–E11856. 30478045 10.1073/pnas.1816189115 PMC6294887

[123] Zhao R, Shen R, Dai H, Perozo E, Goldstein SAN, Molecular determinants of inhibition of the human proton channel hHv1 by the designer peptide C6 and a bivalent derivative, Proc. Natl. Acad. Sci 119 (23) (2022) e2120750119. 35648818 10.1073/pnas.2120750119 PMC9191634

[124] Zhao R, Lopez B, Schwingshackl A, Goldstein SAN, Protection from acute lung injury by a peptide designed to inhibit the voltage-gated proton channel, iScience 26 (1) (2023) 105901. 36660473 10.1016/j.isci.2022.105901 PMC9843441

[125] Tang D, Yang Y, Xiao Z, Xu J, Yang Q, Dai H, Liang S, Tang C, Dong H, Liu Z, Scorpion toxin inhibits the voltage-gated proton channel using a Zn(2+)-like long-range conformational coupling mechanism, Br. J. Pharm (2020). 10.1111/bph.14984 PMC7174885 31975366

[126] Alabi AA, Bahamonde MI, Jung HJ, Kim JI, Swartz KJ, Portability of paddle motif function and pharmacology in voltage sensors, Nature 450 (7168) (2007) 370–375. 18004375 10.1038/nature06266 PMC2709416

[127] Zhang ZJ, Zheng XX, Zhang XY, Zhang Y, Huang BY, Luo T, Aging alters Hv1-mediated microglial polarization and enhances neuroinflammation after peripheral surgery, CNS Neurosci. Ther (2019). 10.1111/cns.13271 PMC7053237 31774629

[128] Pang H, Wang X, Zhao S, Xi W, Lv J, Qin J, Zhao Q, Che Y, Chen L, Li SJ, Loss of voltage-gated proton channel Hv1 decreases insulin secretion and leads to hyperglycemia and glucose intolerance in mice, J. Biol. Chem (2020). 10.1074/jbc.RA119.010489 PMC7076216 31949049

[129] Cao X, Yan L, Hong L, Ion channel mutations and cancer, Biochem Biophys. Rep 42 (2025) 101990. 40236296 10.1016/j.bbrep.2025.101990 PMC11999617

[130] Shen H, Li Z, Jiang Y, Pan X, Wu J, Cristofori-Armstrong B, Smith JJ, Chin YKY, Lei J, Zhou Q, King GF, Yan N, Structural basis for the modulation of voltage-gated sodium channels by animal toxins, Science 362 (6412) (2018). 10.1126/science.aau2596 30049784

[131] Wu X, Li Y, Hong L, Effects of mexiletine on a race-specific mutation in Nav1.5 associated With Long QT syndrome, Front Physiol 13 (2022) 904664. 35864896 10.3389/fphys.2022.904664 PMC9294368

